# In-depth analysis of T cell immunity and antibody responses in heterologous prime-boost-boost vaccine regimens against SARS-CoV-2 and Omicron variant

**DOI:** 10.3389/fimmu.2022.1062210

**Published:** 2022-12-21

**Authors:** Natalie Heinen, Corinna Sophie Marheinecke, Clara Bessen, Arturo Blazquez-Navarro, Toralf Roch, Ulrik Stervbo, Moritz Anft, Carlos Plaza-Sirvent, Sandra Busse, Mara Klöhn, Jil Schrader, Elena Vidal Blanco, Doris Urlaub, Carsten Watzl, Markus Hoffmann, Stefan Pöhlmann, Matthias Tenbusch, Eike Steinmann, Daniel Todt, Carsten Hagenbeck, Gert Zimmer, Wolfgang Ekkehard Schmidt, Daniel Robert Quast, Nina Babel, Ingo Schmitz, Stephanie Pfänder

**Affiliations:** ^1^ Department of Molecular & Medical Virology, Ruhr University Bochum, Bochum, Germany; ^2^ Department of Molecular Immunology, Ruhr University Bochum, Bochum, Germany; ^3^ Center for Translational Medicine and Immune Diagnostics Laboratory, Medical Department I, Marien Hospital, University Hospital of the Ruhr University Bochum, Herne, Germany; ^4^ BIH Center for Regenerative Therapies, Charité-Universitätsmedizin Berlin, Corporate Member of Freie Universität Berlin, Humboldt-Universität Zu Berlin, Berlin Institute of Health, Berlin, Germany; ^5^ Department for Immunology, Leibniz Research Centre for Working Environment and Human Factors (IfADo) at TU Dortmund, Dortmund, Germany; ^6^ Infection Biology Unit, German Primate Center, Göttingen, Germany; ^7^ Institut für klinische und molekulare Virologie, Universitätsklinikum Erlangen und Friedrich-Alexander-Universität (FAU) Erlangen-Nürnberg, Erlangen, Germany; ^8^ European Virus Bioinformatics Center, Jena, Germany; ^9^ Clinic for Gynecology and Obstetrics, Heinrich-Heine-University Düsseldorf, Düsseldorf, Germany; ^10^ Department of Infectious Diseases and Pathobiology, Vetsuisse Faculty, University of Bern, Bern, Switzerland; ^11^ Department of Medicine I, St. Josef-Hospital Bochum, Ruhr University Bochum, Bochum, Germany

**Keywords:** COVID-19, vaccine, immunity, SARS-CoV-2, omicron

## Abstract

With the emergence of novel Severe Acute Respiratory Syndrome Coronavirus-2 (SARS-CoV-2) Variants of Concern (VOCs), vaccination studies that elucidate the efficiency and effectiveness of a vaccination campaign are critical to assess the durability and the protective immunity provided by vaccines. SARS-CoV-2 vaccines have been found to induce robust humoral and cell-mediated immunity in individuals vaccinated with homologous vaccination regimens. Recent studies also suggest improved immune response against SARS-CoV-2 when heterologous vaccination strategies are employed. Yet, few data exist on the extent to which heterologous prime-boost-boost vaccinations with two different vaccine platforms have an impact on the T cell-mediated immune responses with a special emphasis on the currently dominantly circulating Omicron strain. In this study, we collected serum and peripheral blood mononuclear cells (PBMCs) from 57 study participants of median 35-year old’s working in the health care field, who have received different vaccination regimens. Neutralization assays revealed robust but decreased neutralization of Omicron VOC, including BA.1 and BA.4/5, compared to WT SARS-CoV-2 in all vaccine groups and increased WT SARS-CoV-2 binding and neutralizing antibodies titers in homologous mRNA prime-boost-boost study participants. By investigating cytokine production, we found that homologous and heterologous prime-boost-boost-vaccination induces a robust cytokine response of CD4^+^ and CD8^+^ T cells. Collectively, our results indicate robust humoral and T cell mediated immunity against Omicron in homologous and heterologous prime-boost-boost vaccinated study participants, which might serve as a guide for policy decisions.

## 1 Introduction

Vaccines have been a key strategy to contain and mitigate the coronavirus disease 2019 (COVID-19) pandemic, that has – as of November 2022 – claimed over 6.5 million deaths worldwide (1). Currently, over 4.8 billion people have been vaccinated against SARS-CoV-2 infections with different vaccine platforms including but not limited to mRNA (e.g. BNT162b2 (COMIRNATY; Pfizer-BioNTech, Mainz, Germany), mRNA-1273/TAK-919 (Spikevax; Moderna, Massachusetts, USA)) and adenoviral vector-based vaccines (e.g. ChAdOx1 (AstraZeneca, Cambridge, UK)) (1).

Although several lines of evidence indicate that priming and booster vaccination with either mRNA or vector-based vaccines induce humoral immune responses against the ancestral SARS-CoV-2 strain and several variants of concern (VOCs; Alpha (B.1.1.7) (2), Beta (B.1.351) (3) Gamma (P.1) (4) (5), Delta (B.617.2) (6), and Omicron (B.1.1.529) (7)) (8–11), studies have shown that vaccine-induced humoral immunity declines over time, following a first and second dose of BNT162b2 or ChAdOx1 (12, 13). In addition, multiple VOCs and the Omicron variant in particular, have been associated with increased transmissibility and escape from neutralizing antibodies (NAbs) in SARS-CoV-2 vaccinees (14, 15). Ultimately, these observations have led to serious concerns about the longevity and durability of immune memory after vaccination and, more importantly, about the protectiveness against SARS-CoV-2 infections.

Induction of CD4^+^ and CD8^+^ T cell-mediated immunity, another fundamental arm of the adaptive immune system, has been associated with reduced COVID-19 disease severity (16, 17), but is far less well characterized than humoral reactivity to SARS-CoV-2 vaccination. To date, multiple studies have shown that COVID-19 vaccination elicits a stable and fully functional CD4^+^ and CD8^+^ T cell response that is maintained across different vaccine platforms (e.g. mRNA-1237, BNT162b2m Ad26.CoV2.S and NVX-CoV2373) and VOCs (14, 15, 18–26). For instance, boost vaccination with BNT162b2 generated highly differentiated effector CD8^+^ T cells and mobilized a vigorous CD8^+^ T cell response, at times when NAb detection was low (27).

In the past, heterologous vaccination regimens or so-called mix-and-match approaches have been applied due to changing governmental recommendations and have been described to trigger a broader and more robust vaccine-induced immune response (28, 29). Intriguingly, recent data suggests heterologous prime-boost vaccination with BNT162b2 and ChAdOx1 may improve humoral and cell-mediated T cell immunity against SARS-CoV-2 (30–33). Nevertheless, few data are available on the effect of heterologous prime-boost-boost vaccination on T cell-mediated immunity in those who received both vector-based and mRNA vaccines, especially in the context of the currently dominating Omicron variant.

Therefore, we conducted an exploratory longitudinal cohort study of a heterologous and homologous prime-boost-boost vaccination strategy consisting of either combination vaccination with ChAdOx1 and BNT162b2 or triple vaccination with the mRNA vaccine BNT162b2 to compare T cell immune responses against the ancestral SARS-CoV-2 strain and the currently dominant Omicron (B.1.1.529) lineage. We found an activated and memory-like CD4^+^ and CD8^+^ T cell phenotype after both homologous and heterologous prime-boost-boost vaccination. In addition, robust levels of IFN-γ, IL-2, TNF-α and Granzyme B-secreting CD4^+^ and CD8^+^ T cells were detected after SARS-CoV-2 Spike peptide pool stimulation of PBMC from all vaccinees. Overall, homologous and heterologous prime-boost-boost vaccination vigorously recalled both humoral and cellular immune responses against both ancestral SARS-CoV-2 and the Omicron strain.

## 2 Material and methods

### 2.1 Study cohort

Participants were selected based on age (<75 years) and preceding prime vaccination with either a mRNA-based or vector-based SARS-CoV-2 vaccine and planned boost vaccination. The control group was selected according to a homologous vaccination scheme whereas the study group was selected according to a heterologous vaccination scheme.

### 2.2 Study design

**Figure 1:**
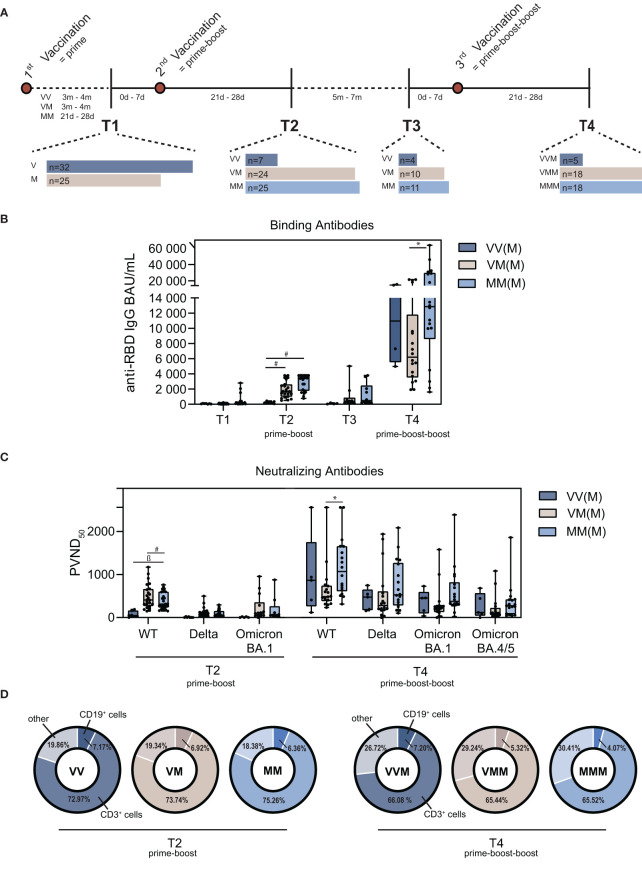
Characterization of the humoral immune signatures upon homologous vs. heterologous prime-boost-boost vaccine regimens. **(A)** Schematic illustration of the vaccination scheme, time periods between vaccinations and PBMC isolation (m=months, d=days). **(B)** Quantification of binding antibody units (BAU) of different vaccine regimens after prime-boost and prime-boost-boost. **(C)** Quantification of neutralizing antibodies as pseudotype virus neutralization dose 50 (PVND_50_) against the wildtype (WT) Spike and variants of concern Delta, Omicron BA.1 and Omicron BA.4/5 after prime-boost and prime-boost-boost vaccination. **(D)** Proportion of CD19^+^ B cells and CD3^+^ T cells in total PBMC population, including NK cells, monocytes and dendritic cells, summarized as other cells. (M = mRNA-based vaccination, V = Vector-based vaccination). Statistical analysis was performed using Tukey’s multiple comparison test (* p=0.05; ǂ p=0.01; ß p=0.001, # p=0.0001).

The study was designed to elucidate the humoral and cellular immunity against SARS-CoV-2 of a heterologous prime-boost and prime-boost-boost vaccination scheme. The study was authorized by the local ethics committee of the Ruhr-University Bochum (21-7260 and 20-6886). After written informed consent, the collected samples were included in this study. For this, whole blood was collected and PBMC and plasma isolated according to previously published studies (34). Initially, we recruited persons with one vaccination (ChAdOx1, vector (V) n=32; BNT162b2, mRNA (M) n=25), which was followed by a homologous prime-boost vaccination (VV n=7; MM n=25) or a heterologous prime-boost vaccination (T2; VM n=24). Samples were collected before another mRNA vaccination (VV n=4; VM n=10; MM n=11) and after another mRNA vaccination (VVM n=5; VMM n=18; MMM n=18). The study consists of four sample collection timepoints: prior and post prime-boost vaccination (T1, T2) and prior and post prime-boost-boost vaccination (T3, T4) (**Figure 1A**).

### 2.3 Virus neutralization assay

Serum was collected from whole blood samples by centrifugation of monovettes at 1500 g for 10 min and stored at -20 °C. SARS-CoV-2 neutralizing antibodies were determined using propagation-defective vesicular stomatitis virus (VSV) pseudotypes and the virus neutalization assay was performed as previously described (35). Briefly, BHK-G43 cells were treated with mifepristone to induce the expression of the VSV Glycoprotein (G) on the surface. Afterwards, cells were incubated with VSV*ΔG(FLuc) and trans-complemented with the G protein (VSV*ΔG(FLuc)+G). The virus particles were used to produce pseudotype virus complemented with the SARS-CoV-2 Spike (S) protein. Therefore, HEK 293T cells were transfected with the appropriate SARS-CoV-2 S expression plasmid for the wildtype (YP_009724390.1) and the VOCs Delta (EPI_ISL_1921353) and Omicron (BA1: EPI_ISL_6640919; BA4/5: EPI_ISL_11550739/EPI_ISL_12029894). After inoculation of the transfected cells with VSV*ΔG(FLuc)+G, the virus particles harbor the respective Spike protein on their surface. To determine the pseudotype virus neutralization (PVN), patient sera were inactivated at 56 °C for 30 min. Sera were pre-diluted 1:10 and 120 µl per donor were pipetted into the first row of a 96 well cell culture plate in triplicates. A twofold dilution was performed and pseudotype virus was added to each well, followed by incubation for 1 h at 37 °C. The suspension was transferred to Vero E6 cells, previously seeded in a density of 1x10^5^ cells per mL in a 96 well plate and incubated overnight. On the next day, the supernatant was aspirated and the cells were lysed with 35 µL luciferase lysis buffer per well. After freezing to the core and thawing, 20 µL of the lysate were pipetted into microtiter plates and luminescence was measured with a plate luminometer. Finally, the antibody dilution resulting in a luminescence reduction of 50%, representing 50% PVN, was calculated (PVND_50_; lower limit of detection: 20 PVND_50_; upper limit of detection: 2560 PVND_50_).

### 2.4 Anti-RBD antibody measurement

Binding antibody units (BAU/mL) against the receptor binding domain (RBD) of the SARS-CoV-2 Spike from the Wuhan strain amongst the groups were measured as previously described (36). Briefly, 96 well plates were coated with recombinant RBD of SARS-CoV-2 WT spike overnight at 4°C and subsequently blocked with ELISA Diluent (Biolegend) for 1 h at 37 °C. Serum samples were serially diluted, including a negative serum control, anti-S antibody as positive control (Dianova, CSB-RA332450A0GMY), and a calibrator. Pre-diluted samples and controls were incubated on the coated plate for 1 h at 37 °C. After washing, HRP conjugated secondary antibody (goat anti human IgG Fc gamma fragment specific, Dianova) was added and incubated for 1 h at 37 °C. After washing, the plate was tapped dry and incubated with substrate (1 Step Ultra TMB, Pierce) for 5-10 min at RT in the dark until the positive control showed distinct blue staining. The reaction was stopped with 2 M H_2_SO_4_ and absorbance was measured at 450 nm. Normalization was performed by: (sample-negative control)/(calibrator-negative control). The sample dilution was used to calculate sample BAU/mL by fitting hyperbolic curves in GraphPad Prism using the correction factor of the WHO Standard 20/136 measurements, which is defined as 1000 BAU/mL.

### 2.5 T cell analysis

Peripheral blood mononuclear cells (PBMC) were isolated from 30 mL blood per donor using standard Ficoll Hypaque density gradient technique as described previously (37) and frozen at a density of 10x10^6^ cells/mL. For flow cytometric analysis of different T cell subpopulations, 2.5 x 10^6^ PBMCs were stained with the viability dye LIVE/DEAD™ Fixable Blue Dead Cell Stain Kit (L23105, Thermo Fisher) for 30 min at 4°C. Subsequently, PBMCs were incubated with the Fc receptor blocking solution Human TruStain FcX™ (422302, Biolegend) for 15 min at 4°C. Afterwards, surface markers were stained for 15 min at 4°C. Fixation, permeabilization and Foxp3 staining was performed using the Foxp3 staining buffer set (130-093-142, Miltenyi Biotec) according to manufacturer’s recommendations. Further information about antibodies is provided in **Supplementary Table S1**. Samples were measured in a Cytoflex LX (Beckman Coulter) and 1.5-2 x 10^6^ events per sample were acquired. Furthermore, SARS-CoV-2 reactive T cells were determined as previously described (37, 38). Briefly, PBMCs were stimulated in the presence of overlapping peptide pools of the WT and Omicron BA.1 Spike SARS-CoV-2 (JPT Peptide Technologies) for 16 h. Brefeldin A (1 μg/mL, Sigma-Aldrich) was added after 2 h. An unstimulated sample served as negative control and stimulation with staphylococcal enterotoxin B (1 μg/mL, Sigma-Aldrich) as positive control. After stimulation, the cells were harvested and stained with optimal concentrations of antibodies (**Supplementary Table S2**) for 10 min at room temperature in the dark. Stained cells were washed twice with PBS/BSA before preparation for intracellular staining using the Intracellular Fixation & Permeabilization Buffer Set (Thermo Fisher Scientific) according to the manufacturer’s instructions. Fixed and permeabilized cells were stained for 30 min at room temperature in the dark with an optimal dilution of antibodies against the intracellular antigen. Stained samples were acquired immediately on a CytoFLEX flow cytometer (Beckman Coulter), collecting at least 25,000 CD3^+^ events. Quality control was performed daily using the recommended CytoFLEX daily QC fluorospheres (Beckman Coulter). No modification to the compensation matrices was required throughout the study. Antigen-reactive responses were considered positive after the non-reactive background was subtracted, and more than 0.01% were detectable. Negative values were set to zero.

### 2.6 Data analysis and sample size

Statistical analysis was performed using Tukey’s multiple comparison test to compare the vaccine regimens with each other and Šidák’s multiple comparison test to compare WT vs. Omicron for each vaccine regimen (* p=0.05; ǂ p=0.01; ß p=0.001, # p=0.0001) in GraphPad Prism 9.4.1. Flow cytometry data were analyzed with FlowJo™ (Becton Dickinson & Company, version 10.8.0 for unstimulated T cells and version 10.7.1 for stimulated T cells). The gating strategies and representative dot plots are shown in **Supplementary Figure S1**. Binding antibody units were calculated in GraphPad Prism 9.

## 3 Results

### 3.1 Study design and characteristics

Since COVID-19 vaccination is available and different vaccination strategies have been applied, the resulting immune response in different healthy and diseased cohorts has since been investigated (34, 39–43). However, further in-depth analysis of the humoral and cellular immune response can add to the existing knowledge, especially in light of the currently dominant Omicron variant. Thus, our study was designed to assess the immune responses of a cohort upon different vaccine regimens after a second dose (prime-boost) and third dose (prime-boost-boost). In total, 57 participants were recruited (65% male, 35% female), with a mean age of 35 ± 12 years. Subjects were general healthy without any evidence of immune deficiency or chronic diseases. Subjects were assigned into three groups, based on the vaccine strategy (**Figure 1A**). Initially, participants have received prime vaccination with the mRNA vaccine COMIRNATY (BNT162b2, Pfizer-BioNTech, here referred to as “M”) or the vector-based vaccine Vaxzevria (ChAdOx1-S, AstraZeneca, here referred to as “V”) prior to participation in the study and did not report previous SARS-CoV-2 infection. For prime-boost vaccination (second dose), the participants either received a homologous boost of the initial vaccine, or a heterologous boost. All of the participants were further vaccinated with COMIRNATY as prime-boost-boost (third dose), except one participant, who received Spikevax (mRNA-1273, Moderna). Accordingly, our cohort is divided into the three groups VVM, VMM and MMM.

### 3.2 Lower humoral immune response against the Omicron VOC compared to WT SARS-CoV-2

Since the beginning of 2022, Omicron (B.1.1.529) is the dominating variant in Europe (44). To elucidate the humoral immune response after vaccination against Omicron in comparison to the ancestral SARS-CoV-2 wildtype (WT) strain, we analyzed binding and neutralizing antibody levels. Binding antibodies were determined as BAU/mL by ELISA (45). All three groups showed increased levels of anti-SARS-CoV-2-Spike binding antibodies 21d-28d after prime-boost (T2) vaccination that decreased within five to seven months post prime-boost (T3) (**Figure 1B**), which is in line with previous studies (9, 46, 47). Interestingly, homologous MM and heterologous VM vaccinated study participants displayed on median significantly higher binding antibody levels compared to VV vaccinated participants after the first boost (median 3451 BAU/mL [MM] and 1704 BAU/mL [VM] vs. 211 BAU/mL [VV]). In addition, MMM vacinees showed significantly higher binding antibody levels compared to VMM after the second boost (median 12856.5 BAU/mL vs. 6212.5 BAU/mL) (**Figure 1B**). Next, we determined neutralizing antibodies against a replication vesicular stomatitis virus pseudotype, which is known to correlate with the classical neutralization against live virus (35). Similar to binding antibody levels, measurement of pseudo virus neutralizing antibody titers (PVND_50_) against the SARS-CoV-2 WT revealed significantly higher neutralizing antibodies in MM vacinees compared to VV vaccinated study participants on T2, as well as for the MMM group in comparison to the VMM group on T4 (**Figure 1C**; **Supplementary Table S3**). Moreover, the three analyzed vaccine regimens displayed reduced neutralizing capacity against Omicron BA.4/5 compared to the Delta variant at T4. Most importantly, neutralizing capacities against BA.1 were significantly reduced in VM and MM on T2. While neutralization was also significantly reduced in VVM, neutralization capacity against BA.1 and BA.4/5 in the VMM and MMM groups was comparable to SARS-CoV-2 WT at T4 (**Figure 1C**; **Supplementary Table S3**). Furthermore, B cell and T cell populations were analyzed after prime-boost (T2) and prime-boost-boost (T4) vaccination by flow cytometry. The total PBMC population showed constant CD19^+^ B cell levels crucial for antibody secretion on T4 compared to T2 for the vector-primed and -boosted group (VVM), whereas the B cell population was reduced for the vector-primed and mRNA-boosted group (VMM) and the homologous mRNA group (MMM). Interestingly, the proportion of CD3^+^ T cells was reduced on T4 for all vaccine regimens (**Figure 1D**). To conclude, humoral immune responses were induced after first and second booster vaccination independent of the vaccine regimen.

### 3.3 Alterations in regulatory T cell response amongst different vaccine regimens and reduced T_FH_ after prime-boost-boost vaccination

**Figure 2:**
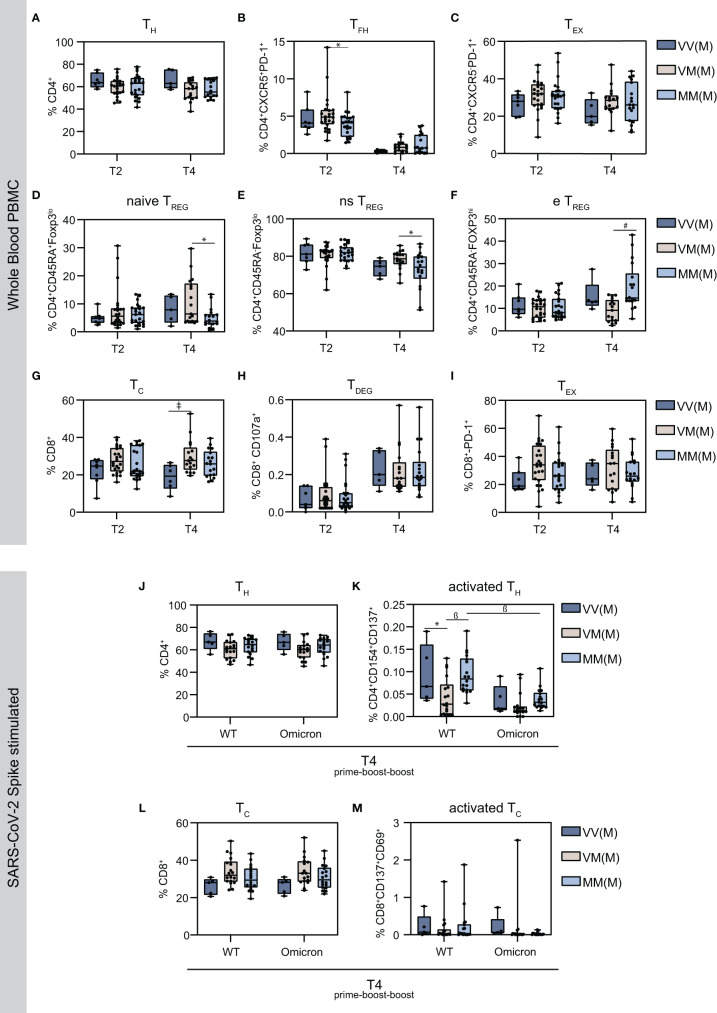
Characterization of the cellular immune signatures upon homologous vs. heterologous prime-boost-boost vaccine regimens. Using flow cytometry analysis, the proportion of the indicated T cell subsets were measured in total PBMC population. **(A)** CD4^+^ T helper cells **(B)** CXCR5^+^PD-1^+^ follicular CD4^+^ T helper cells **(C)** CXCR5^-^PD-1^+^ exhausted CD4^+^ T helper cells **(D)** CD45RA^+^FoxP3^lo^ naïve (ns) regulatory T cells **(E)** CD45RA^-^FoxP3^lo^ non-suppressive (ns) regulatory T cells **(F)** CD45RA^+^FoxP3^hi^ effector (e) regulatory T cells **(G)** CD8^+^ cytotoxic T cells **(H)** CD107a^+^ degranulated cytotoxic T cells **(I)** PD-1^+^ exhausted CD8^+^ cytotoxic T cells **(J)** SARS-CoV-2 S protein reactive T helper cells **(K)** Activated CD4^+^ T helper cells expressing CD154 and CD137 upon stimulation with S protein OPP **(L)** SARS-CoV-2 S protein reactive cytotoxic CD8^+^ T cells **(M)** SARS-CoV-2 S protein reactive cytotoxic CD8^+^ T cells expressing CD69 and CD137. Statistical analysis was performed using Tukey’s multiple comparison test to compare the vaccine regimens with each other and Šidák’s multiple comparison test to compare WT vs. Omicron for each vaccine regimen (* p=0.05; ǂ p=0.01; ß p=0.001, # p=0.0001). The indicated statistics (* p=0.05; ǂ p=0.01; ß p=0.001, # p=0.0001) on the T4 graphics display the comparison to the corresponding T2 for the same vaccine regimen.

T cells are crucial components for effective cellular immunity. They are distinguished into two major subtypes: CD4^+^ T helper cells (T_H_) and CD8^+^ cytotoxic T cells (T_C_). Both subtypes can develop from a naïve, i.e. antigen-inexperienced state, into memory cells that are antigen-experienced and, upon antigen re-encounter, respond with a faster and stronger immune response. Furthermore, follicular T helper cells (T_FH_) and regulatory T cells (T_REG_), both CD4^+^ T cell subsets, are essential for B cell-derived high-affinity antibodies and suppression of over-shooting immune responses, respectively (48–50). To study T cell responses, several T cell subsets were analyzed in whole blood PBMC using flow cytometry and gated accordingly (**Supplementary Figure S1**). First, levels of CD4^+^ cells appeared similar amongst the different groups on T2 and T4, however, the vector primed and boosted group showed a slightly elevated median (**Figure 2A**). Remarkably, the follicular helper T cells (T_FH_) population, characterized by CXCR5 and PD-1 expression, decreased after the second boost compared to the first boost (**Figure 2B**). Moreover, levels of CD4^+^ T cells, which highly express PD-1, a marker of T cell activation and exhaustion (T_EX_) were similar amongst the groups (**Figure 2C**). Regulatory T cells can be subdivided into naïve (naïve T_REG_), non-suppressive (ns T_REG_) and effector T_REG_ (e T_REG_) (**Figures 2D–F**). Interestingly, while levels of naïve and non-suppressive T_REG_ were comparable among the vaccine regimens on T2 and reduced in the MMM group compared to the VMM group on T4 (**Figures 2D, E**), significantly higher amounts of effector T_REG_ were detected on T4 in the homologous mRNA vaccinated (MMM) group compared to the VMM group (**Figure 2F**). In terms of CD8^+^ cells, levels were reduced in the vector primed and boosted group (VVM) on T4, reaching statistical significance in comparison to the heterologous VMM group (**Figure 2G**), only a very small percentage could be identified as degranulated CD107a^+^ cytotoxic T cells (T_DEG_) (**Figure 2H**). No detectable difference was observed in levels of PD-1^+^ exhausted cytotoxic T cells (T_EX_) (**Figure 2I**).

### 3.4 T cell activation markers are more abundant in WT than Omicron spike-stimulated T cells

To specifically analyze SARS-CoV-2-reactive CD4^+^ and CD8^+^ T cells, PBMCs were stimulated with an overlapping peptide pool (OPP) of the SARS-CoV-2 Spike (S) protein. As the original vaccination was developed against the Wuhan SARS-CoV-2 strain (Wuhan-Hu-1, USA_WA1/2020 (51)), the cellular immune reaction against VOCs is of particular interest. To identify possible differences in cellular responses, WT and Omicron BA.1 Spike peptides were selected for stimulation, with Staphylococcal Enterotoxin B serving as positive control. Amongst the different vaccination groups, the proportion of activated CD4^+^ cells was similar for WT and Omicron stimulated PBMCs, however, the activation markers CD154 and CD137 were more abundant upon WT Spike stimulation (**Figures 2J, K**). Moreover, the VMM group displayed significantly decreased levels of activated T cells upon WT Spike stimulation (**Figure 2K**). Comparably, the proportion of CD8^+^ cells was similar amongst the vaccination groups, with lower levels of the activation markers CD137 and CD69 in Omicron Spike stimulated cells (**Figures 2L, M**).

### 3.5 Homologous mRNA vaccine regimen induces higher cytokine production in activated CD4^+^ cells

**Figure 3:**
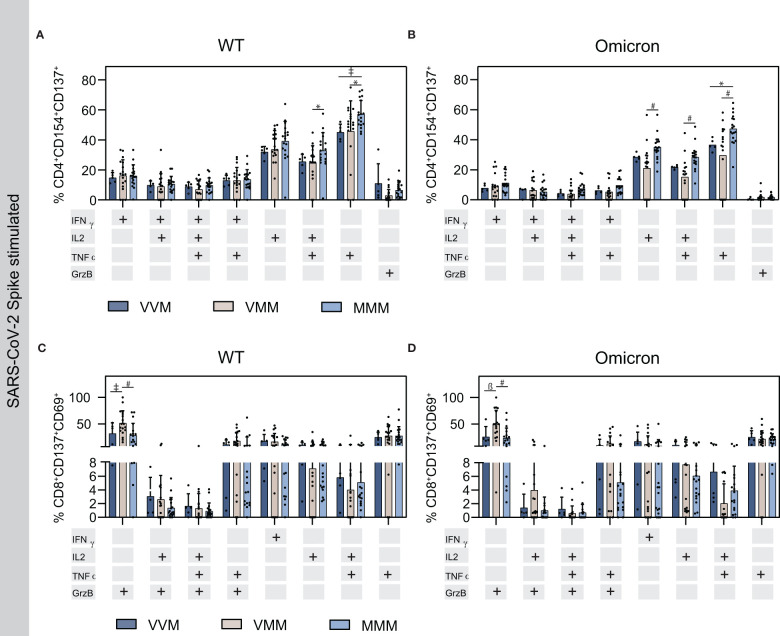
Cytokine production in activated T helper and cytotoxic T cells upon Spike stimulation on T4. **(A, B)** Cytokine production in activated T helper cells upon stimulation with WT **(A)** or Omicron BA.1 **(B)** S protein OPP. **(C, D)** Cytokine production in activated cytotoxic T cells upon stimulation with WT **(C)** or Omicron **(C)** S protein derived OPP. Statistical analysis was performed using Tukey’s multiple comparison test to compare the vaccine regimens with each other and Šidák’s multiple comparison test to compare WT vs. Omicron for each vaccine (* p=0.05; ǂ p=0.01; ß p=0.001, # p=0.0001).

The activation markers CD154 and CD137, or CD137 and CD69 are co-expressed with cytokines in SARS-CoV-2-reactive CD4^+^ or CD8^+^ T cells, respectively. Thus, the production of interferon γ (IFNγ), interleukin 2 (IL-2), tumor necrosis factor α (TNFα) or granzyme B (GrzB) and combinations thereof were measured in activated T cells upon Spike stimulation using flow cytometry and gated accordingly (**Figure 3**; **Supplementary Figure S1**). Interestingly, WT stimulation resulted in a higher percentage of CD4^+^ TNFα^+^/TNFα^+^IL-2^+^ T cells in the homologous MMM vaccine regimen compared to the two heterologous vaccine regimens VVM and VMM, or VMM, respectively (**Figure 3A**). Similarly, Omicron stimulation resulted in a higher percentage of CD4^+^ TNFα^+^/TNFα^+^IL-2^+^/IL-2^+^ T cells in the homologous MMM vaccine regimen compared to VMM and VMM (for TNFα^+^) or VMM (for TNFα^+^IL-2^+^/IL-2^+^) (**Figure 3B**). Notably, this elevated cytokine response upon homologous vaccination complements the observation of increased effector T_REG_ cells in this group to prevent excessive immune reactions compared to the heterologous vaccinations (**Figure 2F**). For the protease GrzB and the cytokine IFNγ, no difference was observed in the different study groups. Similarly, the cytokine production in activated CD8^+^ cells was comparable amongst the vaccine regimens after WT and Omicron Spike stimulation, solely the VMM group displayed significantly higher levels of GrzB^+^ T cells, suggesting increased cytotoxicity of CD8^+^ T cells (**Figures 3C, D**).

### 3.6 T cell memory differs among CD4^+^ and CD8^+^ cell populations

**Figure 4:**
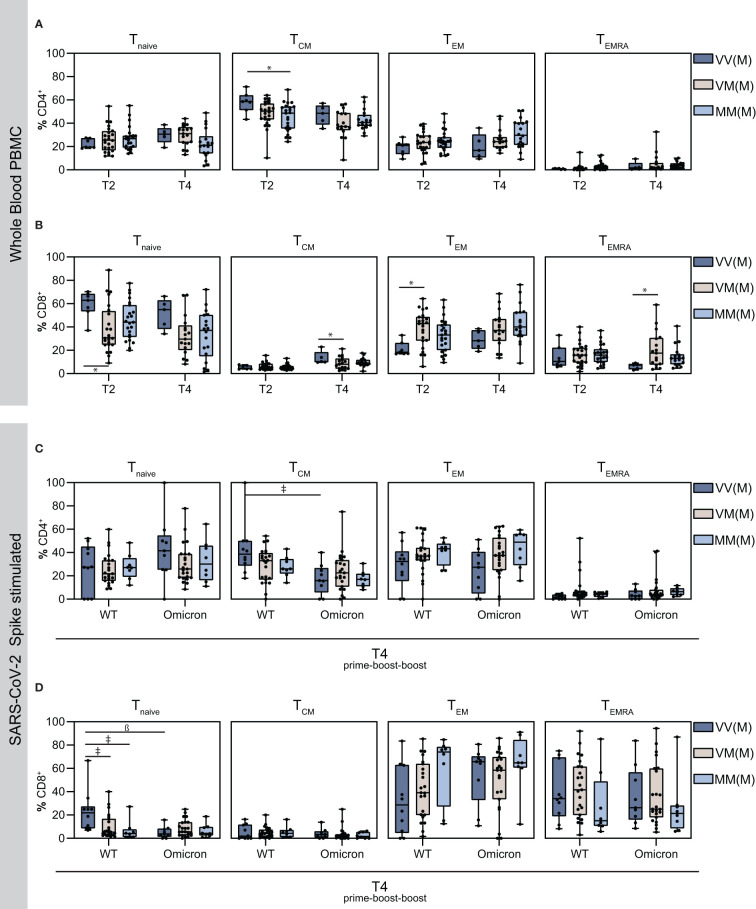
Detection of vaccine-induced T cell memory. Using flow cytometry analysis, the proportion of the indicated T cell subsets were measured in total PBMCs. **(A–D)** naïve, central memory, effector memory and effector memory cells re-expressing CD45RA T cell subsets on T2 and T4 in CD4^+^ T cells **(A)**, CD8^+^ T cells **(B)** and on T4 in Spike-stimulated activated CD4^+^ T cells **(C)** and activated CD8^+^ T cells **(D)**. Statistical analysis was performed using Tukey’s multiple comparison test to compare the vaccine regimens with each other and Šidák’s multiple comparison test to compare WT vs. Omicron BA.1 for each vaccine regimen (* p = 0.05; ǂ p = 0.01; ß p = 0.001, # p = 0.0001).

Responding T cell subsets, including naïve CD4^+^ T cells (T_naïve_), CD4^+^ effector memory T cells (T_EM_), CD4^+^ central memory T cells (T_CM_), and the subset of T effector memory, re‐expressing CD45RA (T_EMRA_), which reside in secondary lymphoid organs (T_CM_), circulate through the blood stream (T_EM_) or exhibit a terminally differentiated phenotype (T_EMRA_), were additionally analyzed in this study (**Figure 4**). Interestingly, CD4^+^ central memory T cells were most abundant amongst the memory cells, with higher frequencies in the VVM vaccine regimen compared to the other groups on T2 and T4, whereas the frequencies of the other responding T cell subsets were comparable among the groups (**Figure 4A**). In contrast, in the CD8^+^ memory cell population, T_EM_ cells were most abundant and the frequency in the VVM vaccine regimen was lower compared to the other groups on T2 and T4. Overall however, the T_naïve_ was the most abundant CD8^+^ T cell subset in VV and MM vaccinees on T2 and VVM vaccinees on T4 (**Figure 4B**). Moreover, after stimulation with S-protein OPP, T cell subsets were analyzed after the second boost, where activated CD4^+^ T_CM_ cells were higher upon WT stimulation compared to Omicron, however, no significant difference was detected between the vaccine regimens except for the VVM group (**Figure 4C**). For CD8^+^ naïve T cells, the VVM group reached significantly higher levels compared to the other groups upon WT stimulation, also compared to the respective Omicron stimulated VVM group. Strikingly, CD8^+^ T_EM_ cells were higher upon Omicron stimulation with the highest in the homologous mRNA vaccinated (MMM) group, whereas the population of T_EMRA_ cells was lower in this group (**Figure 4D**).

## 4 Discussion

As a result of the SARS-CoV-2 pandemic, a considerable effort was made to investigate the safety and efficacy of newly generated vaccines. Recent studies have shown that heterologous and homologous prime-boost vaccine regimens result in adequate humoral and cellular immunity against SARS-CoV-2 (39, 52), and prevent severe COVID-19 disease progression (53). Vaccines were initially developed against the ancestral SARS-CoV-2 Wuhan strain, and studies show that immunity against VOCs, especially Omicron, is equally achieved after the first (15) and second boost vaccination (47) of different vaccine regimens in terms of humoral immunity and rough T cell analysis (14, 47). However, a comprehensive understanding of immune responses and immune memory following vaccination against SARS-CoV-2, including a detailed analysis of immune cells is extremely important to assess vaccine efficacy.

In this study, we first investigated the impact of homologous and heterologous prime-boost-boost vaccination strategies with either mRNA or vector-based vaccines on humoral immunity against the currently dominant Omicron BA.1 and BA.4/5 strains. Evaluation by ELISA and neutralization assay revealed robust vaccine-induced humoral immunity against SARS-CoV-2 WT and VOCs in a young, healthy study cohort with a mean age of 35 years. Notably, in accordance with Tenbusch et al. (31), we found increased neutralization capacities against SARS-CoV-2 WT, Delta and Omicron for heterologous vaccinated participants compared to the homologous group after prime-boost vaccination. Furthermore, in agreement with Beklitz et al., we showed decreased neutralizing capacities against Delta and Omicron in comparison to the SARS-CoV-2 WT (52). These observations are also in line with recent studies that demonstrated increased neutralizing antibody titers after first (prime-boost) (54) and second boost (prime-boost-boost) vaccination (39).

Next, we aimed to investigate cellular immune responses in detail after prime-boost and prime-boost-boost vaccination regimens. Thus, analysis of various cellular T cell subsets was performed in whole PBMC populations to compare the different vaccine regimens. Similarly, cellular T cell subsets were analyzed and studied in SARS-CoV-2 Spike stimulated PBMCs to further compare Omicron BA.1 and WT Spike-reactive T cells. We found that the follicular helper T cells (T_FH_) population significantly decreased after the second boost (T4) compared to the first boost (T2) for all vaccine regimens. T_FH_ are crucial for B cell help in the germinal center and for the generation of high affinity antibodies and activated circulating T_FH_ cells expressing the ICOS and CD38 activation markers were shown to correlate with vaccine responses (55, 56). For long lasting antibody responses, B cells need to maturate in germinal centers, regulated and limited by help of T_FH_ cells (57). As observed in this study, antibody responses measured as neutralizing antibodies, thus representing high-affinity antibodies, are increased on T4 compared to T2. Hence, the reduction of T_FH_ cells in blood could possibly be explained by migration to or retention in germinal centers in lymph nodes and the spleen to facilitate antibody affinity maturation. Studies suggest a T_FH_-mediated immunity in SARS-CoV-2 (58, 59), hence, further investigation of these findings should be considered.

We found more effector T_REG_ cells on T4 in the homologous mRNA-vaccinated group (MMM), compared to both the heterologous groups (VVM, VMM), whereas the naïve T_REG_ population was smaller upon homologous vaccination. Effector T_REG_ are known to suppress other immune cell activities to prevent exaggerated immune responses (60), such as the cytokine storm upon SARS-CoV-2 infection, which is associated with severe and critical COVID-19 manifestations (61). In line with this notion, Xu et al. reported the downregulation of FOXP3, the master transcription factor that determines T_REG_ identity and function, as a cause of secretion of inflammatory cytokines such as IL-6, IL-1 and IL-23 in COVID-19 patients, leading to a decreased number of T_REG_ cells in these patients (62). Hence, it can be speculated that vaccination, especially with mRNA vaccines, results in a protection from an exaggerated immune response, thus emphasizing the safety of vaccination in comparison to natural infection. Supportively, we detected increased levels of IL-2 producing activated CD4^+^ T cells in the MMM group after the second boost on T4, which is known to promote effector T_REG_ differentiation (63, 64). Cytokine producing T cells partly mediate the immune response and were, shown to correlate with the disease progression for natural infections after vaccination for several viral infections (65, 66) including SARS-CoV-2. We detected not only increased levels of IL-2/TNFα, but also TNFα producing activated CD4^+^ T cells following prime-boost-boost vaccination in the MMM group for WT and Omicron, and additionally IL-2 producing CD4^+^ T cells upon Omicron stimulation. Similarly, Schmidt et al. (67) detected increased levels of IL-2 producing activated CD4^+^ T cells for WT in participants with a mRNA boost (VM and MM) compared to a vector boost (VV). This is in line with Gao et al., who reported cross-reactive CD4^+^ T cells against the Omicron variant in individuals receiving two BNT162b2 vaccinations (68). However, it was shown that in particular polyfunctional T cells are responsible for effective immunity (69), thus a potential benefit of the MMM vaccine regimen remains to be elucidated. Interestingly, only differences between WT and Omicron specific activated CD8^+^ T cells were observed for GrzB in the VVM group and no significant differences amongst the other cytokine producing T cells, suggesting similar CD8^+^ immune responses against both variants after three vaccine doses.

Regarding T cell-mediated resolution of viral infections, long-lasting immunity is preserved by T cell memory (70). Hence, analysis of T cell memory in the context of severe COVID-19 is of particular interest (71). T cell memory by natural infection with SARS-CoV-2 results in CD8^+^ T_EM_ and CD8^+^ T_EMRA_ cells (72, 73). CD8^+^ T cell memory to SARS-CoV-2 vaccination results predominantly in CD8^+^ T_EM_ cells (27, 42), which might indicate that vaccination results in less terminally differentiated and, thus, longer lived memory cells. We observed similar findings in our triple vaccinated cohort, in which T_EM_ were the predominant CD8^+^ memory T cell population. Of note, the MMM and VMM vaccine regimes resulted in higher frequencies of T_EM_ and following prime-boost-boost vaccination also in T_EMRA_ CD8^+^ memory T cells. Whether mRNA- or vector-based vaccination leads to greater CD8^+^ T cell immunity is controversially discussed in the literature (reviewed by Sette & Crotty (74)). However, our data suggest that mRNA vaccination is superior in boosting memory T cells responses compared to a second vector-based immunization. Nevertheless, other vector-based vaccines like Sputnik V, might induce similar T-cell responses and were not included in this study.

With respect to CD4^+^ memory cells, SARS-CoV-2 vaccination was reported to result in memory T cells with a T_H1_ and T_FH_ phenotype (reviewed by Sette & Crotty (74)). While we did not investigate the polarization of the CD4^+^ memory compartment, we show that SARS-CoV-2 vaccination primarily induces T_CM_, some T_EM_ and only few T_EMRA_ CD4^+^ memory T cells. Interestingly, homologous vector-based vaccination resulted in higher T_CM_ frequencies, while vaccine regimes with mRNA vaccines resulted in slightly higher T_EM_ frequencies. Regardless, these data warrant future studies to determine which memory T cell subsets correlate with greater immune protection.

In summary, this study elucidated an in-depth analysis of humoral and cellular immunity after different vaccine regimens, currently administered worldwide. We were able to identify several similarities and differences in the immune responses in our healthy and young cohort. These findings contribute to an increased understanding of vaccine-induced immunity and might contribute not only to the education of the general public, but also to health policy measures.

## Data availability statement

The original contributions presented in the study are included in the article/**Supplementary Material**. Further inquiries can be directed to the corresponding authors.

## Ethics statement

The studies involving human participants were reviewed and approved by Ethics committee of the Ruhr-University Bochum (21-7260 and 20-6886). The patients/participants provided their written informed consent to participate in this study.

## Author contributions

Conceptualization, CM, SP. Methodology, SP, IS, NB. Investigation, CM, NH, CB, AB-N, TR, US, MA, CP-S, SB, MK, EB, DU, CW. Writing –Original Draft, NH, MK, SP, IS. Writing –Review and Editing, NH, CM, CB, AB-N, TR, US, MA, CP-S, SB, MK, JS, EB, DU, CW, MH, SPö, MT, ES, DT, CH, WS, DQ, NB, IS, SP. Visualization, NH, JS, SP, DT. Data Analysis, CB, AB-N, DT. Resources SP, MH, SPö, MT, CH, WS, DQ. Supervision, SPa, ES, NB, IS. All authors contributed to the article and approved the submitted version.
