# Supplementary material for: In-depth analysis of T cell immunity and antibody responses in heterologous prime-boost-boost vaccine regimens against SARS-CoV-2 and Omicron variant
Source: Front Immunol. 2022 Dec 21;13:1062210. doi: 10.3389/fimmu.2022.1062210 (PMC9811676; doi:10.3389/fimmu.2022.1062210)
Supplement: Supplementary file 1 [file DataSheet_1.pdf]

Supplementary Material

1 Supplementary Tables

Supplementary Table 1: Antibodies used for flow cytometric analysis of whole blood PBMCs

Specificity	Clone	Fluorochrome	Cat. number	Company	Dilution
CD3	UCHT1	Brilliant Violet 510	300448	Biolegend	1:100
CD4	OKT4	PerCP-Cy5.5	344608	Biolegend	1:500
CD8	RPA-T8	APC- Fire 750	344746	Biolegend	1:500
CD19	HIB19	AlexaFluor 700	302225	Biolegend	1:500
CD45RA	HI100	PerCP	304155	Biolegend	1:100
CD107a	H4A3	Brilliant Violet 711	328639	Biolegend	1:200
CD127	HIL-7R-M21	BUV737	612794	BD Biosciences	1:20
CD185 (CXCR5)	J252D4	AlexaFluor 488	356911	Biolegend	1:100
CD197 (CCR7)	G043H7	APC	353213	Biolegend	1:100
CD279 (PD-1)	EH12.2H7	PE-Dazzle 594	329939	Biolegend	1:20
Foxp3	206D	PE	320107	Biolegend	1:20

Supplementary Table 2: Antibodies used for flow cytometric analysis of SARS-CoV-2 Spike stimulated PBMCs

Specificity	Clone	Fluorochrome	Cat. number	Company	Dilution
Live/Dead	-	LIVE/DEAD Fixable Blue stain	L23105	Thermo Fisher Scientific	-
CD4	SK3	BUV496	612937	BD	1:25
CD8	RPA-T8	V500	560775	BD	1:50
CD69	FN50	FITC	310924	Biolegend	1:100
CD3	Okt3	BV785	317319	Biolegend	1:100
IFNg	4S.B3	BV650	505831	Biolegend	1:50
TNFa	MAb11	eFluor450	48-7349-42	ebioscience	1:100
IL2	MQ1-17H12	PE	500339	Biolegend	1:200
CD137	4B4-1	PE-Cy 7	309817	Biolegend	1:100
GrzB	QA16A02	Alexa Fluor 700	372221	Biolegend	1:200
CD154	24-31	APC-Cy 7	310821	Biolegend	1:200

Supplementary Table 3: Median $PVND_{50}$ values corresponding to Figure 1

Strain	VV[M]	VM[M]	MM[M]	Timepoint
WT	44.91	407.4	311.6	T2
Delta	0	80.91	57.63	
Omicron BA.1	0	100.6	54.2	
WT	864.6	488.95	1069.75	T4
Delta	474.3	277.05	527.05	
Omicron BA.1	453.3	222.45	377.15	
Omicron BA.4/5	117.8	97.93	273.7	

2 Supplementary Figures

Supplementary Figure S1: Gating Strategy used for analysis of T cell subsets via flow cytometry in Whole Blood PBMCs and SARS-CoV-2 Spike stimulated PBMCs. PBMC were incubated for 16 h with overlapping peptide pools (OPP) of the SARS-CoV-2 S-protein from the wildtype strains (WT) and the omicron VOC. Brefeldin A was added 2 h after the OPPs. Untreated PBMC and Staphylococcus aureus enterotoxin B (SEB) as polyclonal stimulus served as negative and positive controls, respectively. Cells were acquired using a Cytoflex LX flow cytometer (Beckman Coulter). Representative gating strategy to identify $CD4^+$ and CD8+ T cells as well as $CD4^+$ $CXCR5^+$ T cells, which share features of T follicular helper cells (T_{FH}) as previously described. Top row from left to right: debris exclusion and lymphocyte identification; cell aggregate exclusion by SSC-H vs SSC-A and FSCH vs FSC-A plots; live/dead (LD) vs. CD3 staining identified live $CD3^+$ T cells; Bottom row, left: $CD4^+$ and $CD8^+$ T cell discrimination; right, $CD4^+$ $CXCR5^+$ T cells (T_{FH}) identification. Identification of T cells reactive against the B.1.1.529 (Omicron)-S-protein among $CD4^+$ T cells (blue frame), $CD8^+$ T cells (red frame), and $CD4^+$ $CXCR5^+$ T cells (black frame).

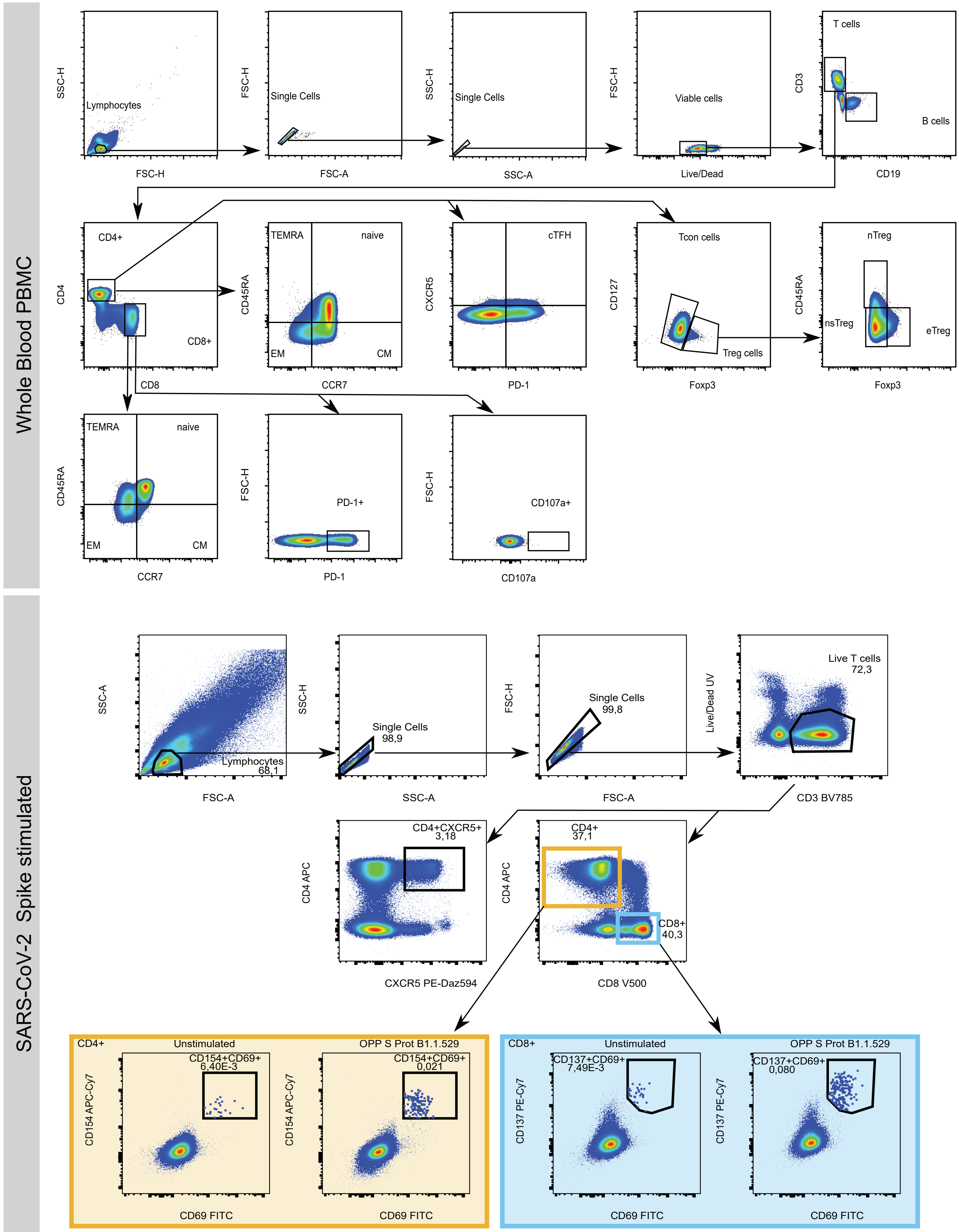
Whole Blood PBMC
SSC-H
FSC-H
Lymphocytes
FSC-A
Single Cells
SSC-A
Viable cells
Live/Dead
T cells
B cells
CD3
CD19
CD4+
CD8+
CD4
CD8
TEMRA
naive
EM
CM
CD45RA
CCR7
cTFH
CXCR5
PD-1
Tcon cells
Treg cells
CD127
Foxp3
nTreg
nsTreg
eTreg
PD-1+
CD107a+
CD107a
SARS-CoV-2 Spike stimulated
Lymphocytes
68,1
Single Cells
98,9
Single Cells
99,8
Live T cells
72,3
Live/Dead UV
CD3 BV785
CD4+CXCR5+
3,18
CD4 APC
CXCR5 PE-Daz594
CD4+
37,1
CD8+
40,3
CD8 V500
CD4+
Unstimulated
OPP S Prot B1.1.529
CD154+CD69+
6,40E-3
CD154+CD69+
0,021
CD154 APC-Cy7
CD69 FITC
CD8+
Unstimulated
OPP S Prot B1.1.529
CD137+CD69+
7,49E-3
CD137+CD69+
0,080
CD137 PE-Cy7
CD69 FITC
